# Effect of shear rate on early *Shewanella oneidensis* adhesion dynamics monitored by deep learning

**DOI:** 10.1016/j.bioflm.2024.100240

**Published:** 2024-11-16

**Authors:** Lucie Klopffer, Nicolas Louvet, Simon Becker, Jérémy Fix, Cédric Pradalier, Laurence Mathieu

**Affiliations:** a Université de Lorraine, CNRS, LCPME, F-54000, Nancy, France; b Université de Lorraine, CNRS, LEMTA, F-54000, Nancy, France; c Unviversité de Lorraine, CNRS, Centrale Supélec, F-57070, Metz, France; d GeorgiaTech Europe, IRL 2958, F-57070, Metz, France; e EPHE, PSL, UMR CNRS 7564, LCPME, F-54000, Nancy, France

**Keywords:** Early bacterial adhesion dynamics, Wall shear rate, Deep learning, Microfluidic system, *Shewanella oneidensis*

## Abstract

Understanding pioneer bacterial adhesion is essential to appreciate bacterial colonization and consider appropriate control strategies. This bacterial entrapment at the wall is known to be controlled by many physical, chemical or biological factors, including hydrodynamic conditions. However, due to the nature of early bacterial adhesion, i.e. a short and dynamic process with low biomass involved, such investigations are challenging. In this context, our study aimed to evaluate the effect of wall shear rate on the early bacterial adhesion dynamics. Firstly, at the population scale by assessing bacterial colonization kinetics and the mechanisms responsible for wall transfer under shear rates using a time-lapse approach. Secondly, at the individual scale, by implementing an automated image processing method based on deep learning to track each individual pioneer bacterium on the wall. Bacterial adhesion experiments are performed on a model bacterium (*Shewanella oneidensis* MR-1) at different shear rates (0 to1250 s^−1^) in a microfluidic system mounted under a microscope equipped with a CCD camera. Image processing was performed using a trained neural network (YOLOv8), which allowed information extraction, i.e. bacterial wall residence time and orientation for each adhered bacterium during pioneer colonization (14 min). Collected from over 20,000 bacteria, our results showed that adhered bacteria had a very short residence time at the wall, with over 70 % remaining less than 1 min. Shear rates had a non-proportional effect on pioneer colonization with a bell-shape profile suggesting that intermediate shear rates improved both bacterial wall residence time as well as colonization rate and level. This lack of proportionality highlights the dual effect of wall shear rate on early bacterial colonization; initially increasing it improves bacterial colonization up to a threshold, beyond which it leads to higher bacterial wall detachment. The present study provides quantitative data on the individual dynamics of just adhered bacteria within a population when exposed to different rates of wall shear.

## Highlights

• Increasing shear rates promote bacterial pioneer colonization up to a threshold , beyond which colonization decreases.
• Deep learning analysis of a *S. oneidensis* early adhered population evidences the dynamic process of nascent biofilms.
• Two shear-dependent bacterial wall lifetimes emerged: dynamic with short residence times and sedentary with longer ones.
• The majority of wall pioneer bacteria adhering were aligned similar or very close to the direction of flow.

## Introduction

The transition of bacteria from a planktonic to a surface-attached state, followed by their adhesion to surfaces, is the fundamental prelude to biofilm formation. Regardless of how one views biofilms [[1], [2], [3]], i.e. (i) as a way of life for collections of individual microbial cells (ii) as main microbial communities with the characteristics of a multicellular organism, or (iii) as structures serving as diversity incubators for microbes, biofilms nevertheless always begin with the initial contact of a planktonic cell with a surface that evolves into structured communities of bacterial cells and embedded into a self-produced extracellular matrix [[4], [5], [6], [7]]. This bacterial entrapment is known to be governed by both molecular and physicochemical interactions as near-wall approaching bacteria experience surface specific interactions, such as hydrodynamic forces, adhesive forces, steric interactions, etc., which govern the adsorption process and their surface dynamics [8,9].

In particular it is well-known that the flow strength plays a predominant role as it determines both the transfer and attachment of planktonic bacterial cells to the surface [1,10,11]. Moreover, hydrodynamic conditions also shape and control mature biofilms as it contributes to the supply of nutrients and oxygen to the biofilm [[12], [13], [14]], the thickness, compactness and 3D-struturation of the biofilm [[15], [16], [17]], the amount and composition of its EPS matrix [[18], [19], [20]] or its erosion [17,21]. Most studies in literature attempt to characterize the influence of hydrodynamics at the biofilm scale, i.e. on a biofilm already established on the surface and defined by a high adherent biomass, surface bacterial growth and EPS production. However, the processes of bacterial adhesion at the very early stages of adhesion under hydrodynamic constraints are less well understood.

Although few data are available, colonization kinetics in the early steps of biofilm formation show that bacterial adhesion is a fast and dynamic process [22,23], making such investigations challenging. Moreover, due to the small number of bacterial events involved at this stage, effective study of early bacterial adhesion requires working with large sample sizes to be statistically robust and at the individual bacterial scale to account for behavioral variability and be representative of the entire pioneering bacterial colonizers.

Microfluidic devices coupled with conventional microscopy techniques (optical, fluorescence or confocal microscopy) benefited to the study of pioneering biofilms in situ, in real time and under controlled flow conditions and are currently the methods of choice for such investigations [[24], [25], [26]]. Numerous strategies, based on both imaging, optical and computational image processing algorithms techniques’ improvement, have been developed to track individual bacteria [[27], [28], [29], [30]]. However, such approaches are facing many technical challenges with respect to instrumental limitations (sensitivity, spatio-temporal resolution, signal to noise ratio), big data processing and analyzing (time-consuming, powerful computational resources and technical expertise needed) or bacteria intrinsic properties (size, motility, bacteria interactions, physiological changes …) [[31], [32], [33], [34]].

To address such challenges, amongst others, deep learning has gained increasing interest in image processing of data collected using various microscopy techniques to detect, segment, track or classify biological objects [29,35,36]. Although this methodology is more established in the case of eukaryotic cells, it is gradually being introduced into the field of microbiology for the study of bacterial cells, as reported in some recent studies. Such applications may include, for instance, the automatic counting and classification of bacterial colonies on Petri dishes [30,37,38], the tracking of bacterial division [39] or the segmentation of bacterial biofilm images [40,41]. However, to our knowledge, this innovative approach has never been introduced to study the behavior of pioneer bacteria in the early stages of biofilm formation.

In this context, our study aimed to evaluate the effect of wall shear rate on the early bacterial adhesion dynamics within a capillary system. Beyond the standard time-lapse approach for assessing bacterial colonization kinetics, we decided to work at the individual scale, by implementing an automated image processing method based on deep learning to track each individual pioneer bacterium at the wall. To achieve this goal, the first 2 h of colonization kinetics of a model bacterium *Shewanella oneidensis* was performed under controlled hydrodynamic strength conditions. We aimed to assess (i) the contribution of each of the transfer mechanisms (convection, sedimentation or diffusion) to the bacterial adhesion, (ii) the influence of the shear rate on the behavior of the pioneer adhered bacteria in terms of residence time and orientation at the wall and (iii) the performance of deep learning approach on early adhered bacteria tracking. We have demonstrated such an original approach applied for the first time to early events colonizing a surface that holds great promise for analyzing massive data.

## Materials and methods

### *Shewanella oneidensis* strain and inoculum preparation

Experiments were performed using *Shewanella oneidensis* MR-1 as a model strain that constitutively expressed the green fluorescent protein (gfp). This strain was constructed by Teal et al. [42] using gfpmut3∗ gene insertion in the *S. oneidensis MR-1* genome using a modified Tn7 delivery system. This strain, stored at −80 °C, was used for all the experiments.

### Microfluidic system and hydrodynamic conditions

**Fig. 1:**
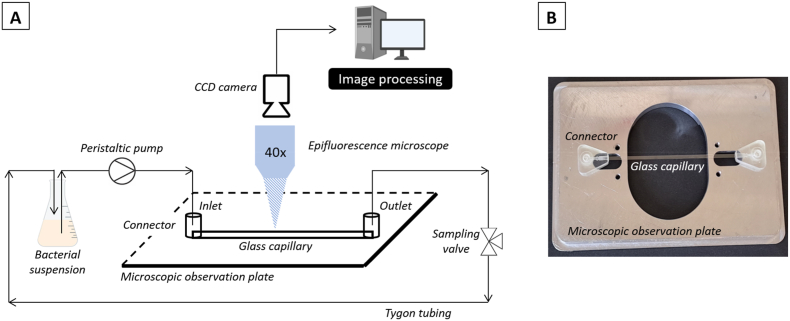
A) Schematic diagram of the closed-loop experimental setup. B) Focus of the microfluidic glass capillary (0.2 × 2 × 100 mm^3^).

Early bacterial dynamic adhesion experiments were studied in a microfluidic flow cell (Fig. 1). A high aspect ratio rectangular glass capillary (Vitrocom, height *h* = 0.2 mm, width *w* = 2 mm, length *L* = 100 mm), was connected to a peristaltic pump or syringe pump depending on the imposed flow rate *Q*. For each assay, a new entire microfluidic device (capillary and tubing) was used and cleaned with the following protocol: SDS (0.1 % - 30 min - 1 mL/min), ultrapure water (30 min at 1 mL/min), HCL (0.1 M - 30 min at 1 mL/min) and ultrapure water (30 min - 1 mL/min).

In order to evaluate the hydrodynamic conditions on the bacterial adhesion the flow rate *Q* was varied from 10 to 1000 μL/min. The shear rate generated at the walls is well approached by the formula [43]:

(1) $${\dot{\gamma }}_{w}=\frac{6Q}{wh²}$$

In the range of applied flow rates, this led to wall shear rates ranging between 13 and 1250 s^−1^, which correspond to wall shear stresses of 0.013 and 1.25 Pa (Table 1). These values are within the range of typical wall shear stress experienced by bacteria in many flow configurations (real or lab-scale) for biofilm formation [15,[44], [45], [46], [47], [48]]***.*** Static condition was also tested as a control. The Reynolds number was calculated for each condition as follows: $Re=\rho {uD}_{h\;}/\eta$ where *ρ* the fluid density (*ρ* = 1000 kg/m^3^) and *η* its dynamic viscosity (*η* = 10^−3^ Pa.s). *u* corresponds to the average fluid velocity and *D*_*h*_ to the device hydraulic diameter with $D_{h}=2wh/\left(w+h\right)$. The Reynolds number was always lower than 15 ensuring laminar flow condition.

**Table 1:** Values of flow rate (*Q*) and associated hydrodynamic parameters of the capillary device: wall shear rate (${\dot{\gamma }}_{w})\text{,}$ wall shear stress (*σ*_*w*_), Reynolds number (*Re*), average fluid velocity (*u*) and estimated and ratio of characteristic times of mass transfer: hydrodynamic residence time (*T*_*r*_), sedimentation time (*T*_*s*_) and diffusion time (*T*_*d*_).

*Q* (μL/min)	10	100	300	500	1000
${\dot{\gamma }}_{w}$ (s^−1^)	13	125	375	625	1250
*σ*_*w*_ (Pa)	0.013	0.125	0.375	0.625	1.25
*Re*	1.5 10^−1^	1.5	4.5	7.6	15.2
*u* (mm.s^−1^)	4.2 10^−1^	4.2	12.5	20.8	42.0
*T*_*r*_ (s)	240	24	8	4.8	2.4
*T*_*s*_ (s)	500	500	500	500	500
*T*_*d*_ (s)	8300	8300	8300	8300	8300
*T*_*d*_*/T*_*r*_	35	346	1038	1729	3458
*T*_*d*_*/T*_*s*_	17	17	17	17	17
*T*_*s*_*/T*_*r*_	2	21	63	104	208

### Early bacterial adhesion experimental setup

Prior to assay, *S. oneidensis* from the glycerol stock at −80 °C was recovered onto Lysogeny Broth Lennox (LB, Sigma-Aldrich L3022) agar plates. After 2 days incubation at 30 °C, several isolated colonies of *S. oneidensis* were used to inoculate a 250 mL Erlenmeyer flask containing 50 mL of sterile 10-fold diluted LB medium (LB_1/10_). After this overnight preculture (30 °C, 160 rpm) the bacterial suspension on stationary growth phase was centrifuged (4375 g for 4 min) and resuspended in fresh LB_1/10_ medium to reach a final concentration close to 5 × 10^7^ ± 3 × 10^6^ cells/mL. Prior to inoculation, a conditioning step [[49], [50], [51], [52]] with LB_1/10_ medium was performed for 2 h at 100 μL/min. The microfluidic system (Fig. 1), was then inoculated with the bacterial suspension in a closed loop for 2 h at a constant flow rate depending on the experimental conditions (Table 1). The time T_0_ of the test corresponds to the moment when the entire system was filled with the suspension and indicates the start of the 2 h test. A minimum of 3 independent experiments per shears were conducted and averaged.

### Quantification of planktonic *S. oneidensis* planktonic by culture and flow cytometry

To study the evolution of *S. oneidensis* concentration during the adhesion experiments, regular samples were taken directly from the flask containing the inoculum. On each of them, measurements of culturability by plating and total gfp bacteria by flow cytometry (FCM) were done. For culturability, 10 μL from multiple 10-fold dilutions of the bacterial suspension were plated on LB_1/10_ agar media and after incubation of 2 days at 30 °C, the number of colonies were counted and results expressed as CFU per milliliter. For total bacteria counting, analysis was performed with the BD Accuri™ C6 Plus flow cytometer (BD Biosciences, USA) equipped with a blue laser at 488 nm. Ultrapure water (OTEC Aguettant, France) was used as sheath fluid. GFP-bacteria were detected using the FL1 detector (533 nm ± 30). Events were triggered on the forward scatter (FSC) parameter with a threshold 2500 and on the FL1 detector with a threshold of 700. Flow cytometer data were acquired on diluted sample (1:10 in ultrapure water, OTEC Aguettant, France) over 2 min at a flow rate of 35 μL/min and were analyzed using BD Accuri™ C6 software (BD Biosciences).

### Quantification of adhered *S. oneidensis* by microscopy and time-lapse processing

Pioneer adhesion was studied using video microscopy. The microfluidic system was mounted under an epifluorescence microscope (Zeiss Axioskop, Germany, 40x/0.6 objective) in a temperature-controlled chamber where an average temperature of 22.4 °C ± 0.3 °C was maintained. The system was equipped with a CCD camera (P830C 8.3 MPixels) recording at 0.5 frames per second and time exposure of 800 ms. Images were taken at the center of the channel on the upper or lower capillary wall with an observation field of approximately 550 × 310 μm^2^ (previous assays evidenced a homogeneous bacterial colonization throughout the capillary length as shown in Fig. S1). Bacteria were considered adhered if they were immobilized at the capillary wall defined by the focal plane of the microscope.

For the time-lapse processing, due to a GFP photo-bleaching of *S. oneidensis* for times exceeding 15–20 min, the observation zone was moved every 14 min to the next microscopic field in the direction of flow. This means that the time-lapse process consisted of successive videos of 14 min each, over a continuous period of 2 h. Two types of image analysis were then carried out: one for the entire 2 h period, using conventional manual counting of adhered bacteria, and the other for the first 14 min of the experiment, using deep learning. The following sections describe these approaches.

### Image analysis by manual counting

Images captured over 2 h were processed with ImageJ to enhance image contrast and brightness of the gfp-bacteria fluorescence. Adhered bacteria were manually counted on a panel of 85 representative images taken all along the 2 h. The concentration at the capillary surface was determined by dividing the number of bacteria counted by the observation zone area (1.7 10^−3^ cm^2^).

### Deep learning detection and tracking algorithms and parameterisations

The detection of bacteria over the initial 14 min of experiments was achieved using YOLOv8, a state-of-the-art object detection neural network [53,54]. YOLOv8 is a convolutional neural network that is specifically designed for object detection which means, in our study, predicting the bounding boxes around every individual bacterium. The following 3-steps process was used to finally obtained the position of each bacterium: label bacteria to build the training dataset, train the neural network model on the dataset and run the model for bacteria detection (inference).

The labelling step was done with the labelbox web-plateform software (Labelbox. Available online. https://labelbox.com). Labelbox is a data management and annotation platform used for developing and training machine learning models. Bacteria were framed on full resolution images (3160 × 2180 pixels) to build the dataset. To ensure that the "bacteria" class was representative, images were selected from different hydrodynamic conditions and at different colonization times. Due to the inherent variability in bacterial size, fluorescence intensity and noise level, we applied an iterative approach to enhance model detection performance by incorporating new image samples in the dataset. This iterative improvement was done by training YOLO on the new expand dataset starting from the previous weight configuration file and then detecting bacteria on the whole film database. This operation was repeated until that most of bacteria were detected and satisfactory metrics were obtained (see below). The final dataset was composed of 300 images and more than 30,000 labelled bacteria.

The second step was to train the model by using the smaller architecture size YOLOv8n (which define the number of trainable parameters). For memory management purposes (see below for the detection step) full resolution images were divided on 256 × 256 pixels crops associated with labels. The model has been pre-trained on the COCO database [55], that fix the initial weights, and then finetuned on our labelled dataset. The training fold accounts for 80 % of the total dataset and 20 % for the validation fold. The training of YOLOv8 used the default parameter values as defined by ultralytics. It involves various data augmentation techniques to improve generalization of the trained network such as vertical flip, random translation, rotation, zoom, mixup … After some trials, the model was finetuned over 100 epochs with confidence *c* = 0.3 and an Intersection of Union *IoU* = 0.7. The confidence is the threshold on the predicted objectness and decides which predicted bounding boxes are kept for the final prediction. The *IoU* is used to merge overlapping bounding boxes in the Non-Maximum Suppression step of YOLOv8. The implementation of ultralytics involves early stopping the validation loss to select the best model. The recall and precision metrics for the best model were respectively *R* = 0.78 and *P* = 0.78.

**Fig. 2:**
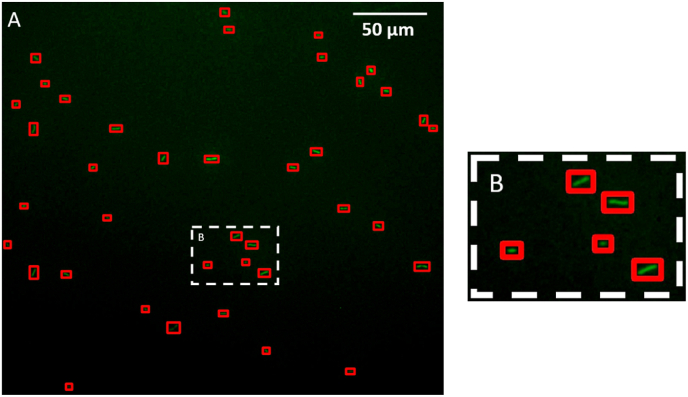
A) Detection result obtained with the trained model used. It detects all adhered *S. oneidensis* gfp cells at the wall and frames them with a bounding box. B) corresponds to a zoomed section of image A. Movie 1: Example of bacterial detection and tracking result obtained during the first 14 min of pioneer bacterial colonization at the capillary wall with the trained model used. We observe 1) the detection of all bacteria arriving at the wall and 2) the associated tracking along at the wall. The color and color variability of the bounding boxes surrounding is a result of the Kalman filter operation. Flow is from right to left. Video dimensions is 550 μm × 310 μm.

The detection (inference) step was performed using SAHI (Slicing Aided Hyper Inference) a tool devoted to improve small object detection and integrated with YOLOv8 [56]. Indeed, a medium GPU (Nvidia RTX 3000 with 6 GB memory) has not enough memory to process 1 full frame image (3160 × 2180 pixels) even with the smaller architecture YOLOv8n. YOLOv8 gives the possibility to downscale images before processing to reduce memory consumption which works fine for detecting quite large objects. However, in our experimental configuration, bacteria was too small to be downscale and lead to poor detection level. To overcome this problem, SAHI slices the trained objet detector over the full-sized images, performing inference on crops (256 × 256 sub-images) and then merges the predictions to obtain full-frame predictions. Bacterial detection using the trained model is illustrated in Fig. 2.

Eventually, the individual dynamic of each bacterium was deduced from a tracking step which was performed by a Kalman filter. Such filter tracks bacteria along consecutives frames [57] as shown in Movie 1 which is a representative example of bacteria tracking obtained at the wall.

### Quantification of bacterial residence time and orientation at the wall

Detection and tracking obtained from the deep learning model and Kalman filter analysis enabled us to measure the wall residence time of each recorded bacterium that adhered to the wall during the acquisition period (14 min), i.e. the time that the bacterium remained at the wall, by subtracting the bacteria's arrival time at the wall from its departure time. Bacteria that did not leave the wall during the analysis period (less than 9 % of the total population), were assigned with a residence time equal to the final video time minus the bacteria arrival time.

In addition, each bounding box detected during the acquisition period was post-processed to calculate the most likely wall orientation of the bacteria. An average wall angle, relative to the flow direction, was assigned to each tracked bacterium by averaging all the angle it took during its wall residence time. These two parameters were automatically determined using a MATLAB program for all bacteria detected at the wall in this study, over 20,300 bacteria.

### Statistical analysis

Statistical analyses were performed using XLSTAT 2023–1.5 software (https://www.xlstat.com/fr). The Spearman correlation test was performed to assess the agreement between what the implemented neural network detects and conventional microscopic counting. The data sets between the upper and lower bacterial wall colonization levels for each shear rate tested were compared using the Mann-Whitney test, a non-parametric test of the null hypothesis that two independent samples came from the same population. The Kruskal-Wallis test, a non-parametric test of the null hypothesis that all samples were from the same population, was performed to assess whether shear rates had an effect on both the upper bacterial wall colonization and bacterial wall residence time. Data sets were considered significantly different if the p-value was below 0.05.

## Results

### *S. oneidensis* inoculum characteristics in the microfluidic system

The bacterial inoculum at the beginning of the adhesion experiment contained an average of 4.6 × 10^7^ bacterial cells/mL (Table 2), among which approximately 98 % are non-permeable to propidium iodide (Table S1) and 8 % were culturable. Despite a slight non-significant (p = 0.060) improvement in culturability at *T*_*2h*_, no significant increase (p = 0.296) of the total bacterial biomass quantified by flow cytometry is observed, indicating no measurable planktonic bacterial growth in the experimental system along the 2 h of adhesion-step. This also suggests that the bacterial colonization of the capillary wall, is driven primarily by bacterial attachment and not by *S. oneidensis* growth in the planktonic phase.

**Table 2:** Bacterial inoculum characteristics: number of total bacteria (cells/mL) and culturable bacteria (CFU/mL, 48h à 30 °C) in the feeding medium (planktonic form) at the beginning (t = 0h) and the end of the bacterial adhesion's phase (t = 2h). All the data represent mean and standard error from 18 independent experiments.

Time (h)	Total bacterial cell (cells/mL)	Culturable bacterial cell (CFU/mL)
0	4.6 × 10^7^ ± 2.5 × 10^6^	3.5 × 10^6^ ± 2.2 × 10^5^
2	4.2 × 10^7^ ± 2.5 × 10^6^	5.5 × 10^6^ ± 6.0 × 10^5^

### Bacterial wall transfer mechanisms relative to shear rates

Values of flow rates and associated hydrodynamic parameters are presented in Table 1. Three passive mechanisms can be considered to explain bacteria mass transfer from the bulk liquid environment to the wall [9,58]: convection (governed by liquid flow velocity), sedimentation (governed by gravity) or diffusion (governed by thermal motion).

Characteristic times associated with these mechanisms were estimated for each of them. The hydrodynamic residence time $T_{r}=L/u$, linked to convection, is the time it takes for bacteria to cross the length of the capillary *L* at the average velocity *u*. The bacteria sedimentation time $T_{s}=h/\left(2V_{s}\right)$, with *V*_*s*_ the bacterial sedimentation velocity, is the time it takes for bacteria to reach the wall due to gravity considering a characteristic distance to travel equal to half the capillary height *h/2.* The sedimentation velocity was estimated through the stokes law $V_{s}=2R²g\Delta \rho /9\eta \approx$ 2.10^−7^ m/s considering bacteria density close to 1088 kg/m^3^ and a size *R* ≈ 1 μm [59]. In the same way, the diffusion time is defined as $T_{d}=\left(h/2\right)²/6D\text{,}$ the characteristic time needed for bacteria to reach a wall by Brownian motion with *D* the bacterial diffusion coefficient in water estimated as 2.10^−13^ m^2^/s by the Stokes-Einstein law D=kT/6πηR [8,60].

The ratios between these different times provide a means of comparing the relative contribution of each of these transfer mechanisms, depending on hydrodynamics. Except for static condition, for all shear rate tested, diffusion could be neglected as the diffusion time was at least 17 times greater than hydrodynamic residence or sedimentation times. A comparison of hydrodynamic residence time and the sedimentation time revealed that for high shear rate (as soon as ${\dot{\gamma }}_{w}$ ≥ 125 s^−1^) convection was the main mechanism responsible of bacterial wall transfer as sedimentation time was at least 21 times greater than hydrodynamic residence time. One the contrary, at low shear rate, sedimentation time and convection time were of the same order of magnitude (the difference is less than a factor 2 for shear rates below 13 s^−1^). Therefore, for weak hydrodynamics, gravitational sedimentation has to be considered in such confined system.

**Fig. 3:**
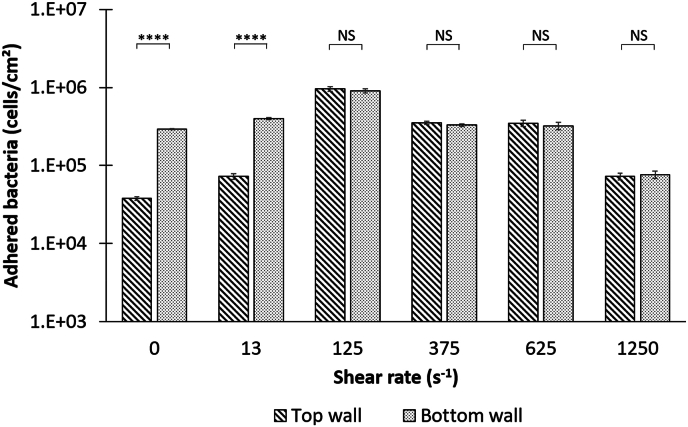
Concentration of adhered *S. oneidensis* at the top and bottom wall of the capillary according to shear rates after 2h colonization. For each shear rate, histograms represent mean and standard error for 30 independent values per shear rate and per wall condition. (NS = not statistically significant, ∗∗∗∗ = p value < 0.0001).

To experimentally evaluate the contribution of such a mechanism to bacterial transport and wall deposition under shear rates, we compared the top and bottom colonization obtained after 2 h in our experimental device obtained (Fig. 3). Once it was confirmed that the capillary surface was uniformly colonized throughout its entire length (Fig. S1), the results demonstrated that there was no significant difference in the top and bottom colonization for shear rates greater than 125 s^−1^ (respective p-values of 0.406 for ${\dot{\gamma }}_{w}=$ 125 s^−1^, 0.398 for ${\dot{\gamma }}_{w}=$ 375 s^−1^, 0.449 for ${\dot{\gamma }}_{w}=$ 625 s^−1^ and 0.767 for ${\dot{\gamma }}_{w}=$ 1250 s^−1^). This indicate that the colonization observed was independent of sedimentation. However, in the absence of shear forces and for the lowest shear rate (13 s^−1^), the concentration of adhered bacteria was significantly impacted by sedimentation, with over 87 % of the bottom wall colonization attributable to this wall transfer mechanism (Fig. 3). As observed experimentally and subsequently confirmed in more theoretical terms, the wall transfer of bacteria in our one-pore confined system is dependent on the shear rates and can be attributed to either convection or sedimentation. Bacterial motility may also be involved, as observed previously [[61], [62], [63]]. *Shewanella oneidensis MR-1* is known to carry a set of genes for the expression of a single unsheathed polar flagellum [64,65], giving it the ability to move in its environment when it is expressed. Such expression is highly regulated [66] and environmental factor dependent [[67], [68], [69], [70], [71], [72]]. In our case, motility measurements done in static condition (data not shown) indicate that approximately 10 % of the bacterial inoculum was motile at the beginning of the experiment. However, under shear rates, motility transfer at the wall is evidenced to be low or even negligible compared to convection transfer. Indeed, the swimming speed of *Shewanella oneidensis*, between 25 and 60 μm/s [67,73], is 1–3 orders of magnitude less important than the capillary average fluid velocity, which is between 42 × 10^1^ and 42 × 10^3^ μm/s depending on the experiments (Table 1). A deeper analysis of the balance between motility and the shear flow can be found in supplementary data S1 and confirmed that motility was not predominant in this study.

The remainder of this study will exclusively focus on the top wall bacterial colonization, where solely convection occurred. Fig. 3 also illustrates that shear rate influences the level of bacterial colonization, a finding that will be analyzed and discussed in greater detail in the subsequent section.

### Effect of shear rate on the early colonization kinetics of *S. oneidensis*

**Fig. 4:**
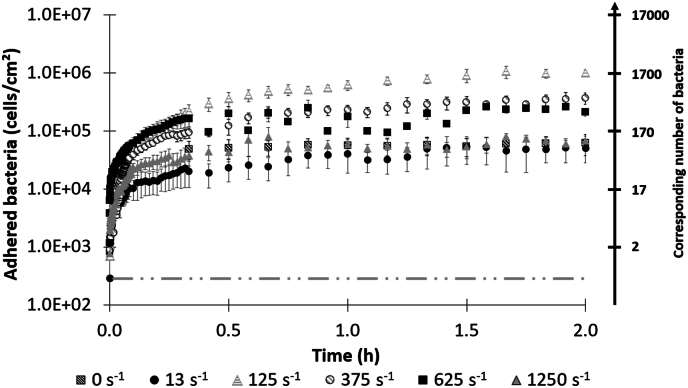
Temporal evolution of pioneer adhered *S. oneidensis* (cells/cm^2^) at the capillary upper wall for different shear rate as a function of time. Left axis: adhered bacteria surface concentration. Right axis: equivalent number of bacteria detected within the microscopic observation zone. Each point is the mean of three independent experiments. Error bars represent the standard error. The dotted line indicates the detection limit (1 bacterium in the observation zone).

Bacterial colonization kinetics are obtained using time-lapse images and manual counting (Fig. 4). A two-phase colonization is observed with a first phase of fast bacterial deposition, followed by a slowdown phase suggesting that bacteria absorption and desorption events balance each other [8,58]. Indeed, in all conditions tested, the concentration of bacteria adhering increased with time and their pseudo-plateau values were significantly dependent on the shear rates (p < 0.0001). In particular, a higher level of colonization was obtained at a shear rate of 125 s^−1^ (10^6^ cells/cm^2^ after 2 h), corresponding to 9 % of wall coverage (Fig. S2). In contrast, the lowest levels of colonization are obtained at the lowest (0 and 13 s^−1^) and highest (1250 s^−1^) shear rates tested, where the concentration of adhered bacteria is about 13 times lower and represents less than 2 % of top wall coverage (Fig. S2). Representative images of the colonization evolution of the upper surface over 2 h for ${\dot{\gamma }}_{w}=$ 125 s^−1^ and ${\dot{\gamma }}_{w}=$ 1250 s^−1^ are shown in Fig. S3.

In addition, initial colonization rates varied non-linearly with the applied shear rate, i.e. the rate and the level of colonization are not associated with greater hydrodynamic stress (Fig. S4). To our knowledge, few studies have measured initial colonization rates over such short adhesion times [23,74,75]. They obtained similar findings, although the strains, hydrodynamic conditions and surfaces to colonize were different. Besides, except for ${\dot{\gamma }}_{w}=$ 125 s^−1^, a higher colonization rate is observed during the 20–30 first minutes of colonization, between 10^3^ and 10^4^ cells.cm^−2^.min^−1^, followed by a slowing down of the colonization rates (reduction factor from 2 to 13 depending on the shear rates), leading to a pseudo-plateau. Several hypotheses could explain such a slowdown rate. Firstly, as the colonizable surface that bacteria encounter evolved over time, from a “blank” surface at the beginning to a progressively colonized surface, the interaction sites available at our pre-conditioned glass surface became saturated gradually as bacteria deposited. However, this possible explanation is not consistent with our data showing a low level of coverage (10 % at best; Fig. S2). Besides, when bacteria reached the wall, they may have collided with bacteria already adhered, which may have helped or prevented them from attaching to the wall and led to the detachment of initially attached bacteria [76,77]. Second, one bacterium already attached could also interfered with the adhesion of another due to hydrodynamic blocking [78,79]. This phenomenon considers that adhered bacteria exhibited a “blocking area”, corresponding to a “shadow zone” around them that may prevent further bacteria from arriving and attaching in this area. Ko and Elimelech [79] proposed that this area was determined by the combined effect of hydrodynamic interaction: the shear component of the fluid flow around adhered particles, and electrostatic repulsion between the adhered and incoming bacteria. Already studied in the case of colloidal particles [79,80] and bacteria cells [8,79,81,82], it has been demonstrated that the size of this blocking area depends on the ionic strength of the media, the surface (charge, hydrophobicity) and cell properties (size, surface appendages), but also and more interesting in our case, to increase with the shear rate. This phenomenon has been described as contributing to the limitation of bacterial colonization and the establishment of a pseudo-plateau, leading to very low coverage rates (close to 10 %) [58].

Most importantly, our results indicate the absence of a linear relationship between shear rate and pioneer colonization, as we obtained a notably bell-shaped profile between shear rates and the bacterial concentrations at 2 h (Fig. 3). Thus, increasing shear initially improved bacterial colonization and its rate (Fig. 4 and S4) until an optimum shear was reached (in our case close to ${\dot{\gamma }}_{w}=$ 125 s^−1^), beyond which increasing shear had the opposite effect. Such bell-shaped profile has already been observed [10,11,22,46,83]). Indeed, Busscher and Van Der Mei [8] and Park et al. [11] showed that increasing shear rate improved bacterial transport, leading to an increased probability of collision of bacteria with the wall. Above an optimal threshold, the shear rate led to an increased probability of bacteria detachment from the wall, because shear became stronger than the interaction forces that bacteria could have developed with the wall.

### Validation of the deep learning analysis

**Fig. 5:**
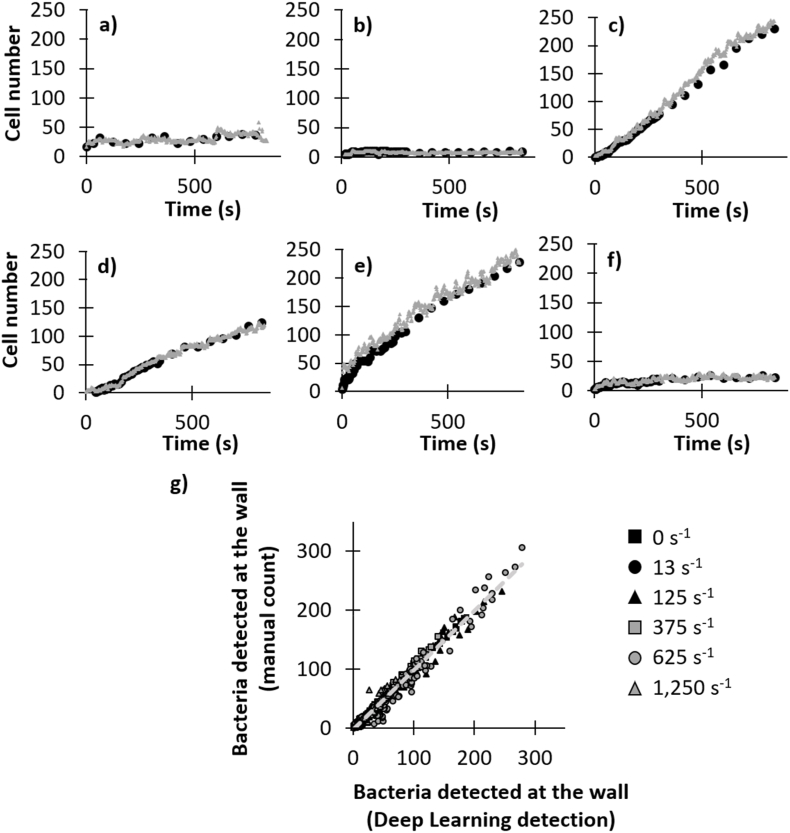
Graph (a), (b), (c), (d), (e) and (f) are representative examples of wall colonization kinetics by GFP-tagged *Shewanella oneidensis* MR-1 for the different shear rates tested (a = 0 s^−1^, b = 13 s^−1^, c = 125 s^−1^, d = 375 s^−1^, e = 625 s^−1^, f = 1250 s^−1^): bacteria detected by manual counting (black circle) and bacteria detected by deep-learning detection (grey triangle). (g) Correlation between the number of bacteria detected either by manual counting or by the deep learning model for all the tests and shear rates tested. The data set comprises 706 images with nearly 28,000 detected bacteria.

Prior to implementation, a validation step to ascertain the effectiveness of what is detected by the neural system compared to conventional microscopic counting was conducted. Fig. 5 (a, b, c, d, e, f) illustrates the representative colonization kinetics at the capillary wall obtained through both manual counting and deep learning detection under varying different shear rate conditions. There is a strong correlation between the results obtained from these two enumeration methods, irrespective of the shear rate under consideration or the level of colonization attained. Therefore, both manual counting and deep learning model detection of bacteria yielded comparable outcomes as observed in Fig. 5(g) (R^2^ = 0.969; Spearman correlation test p-value <0.0001). The discrepancy between these two methods, regardless of colonization time, assay and shear rate, exhibited a total error percentage of 6 % and an average absolute difference of 5 bacteria (with a standard deviation of ±0.3). This validates the use of the YOLO model for the detection of the initial stages of bacterial adhesion to the capillary wall.

### Bacterial residence time at the wall

**Fig. 6:**
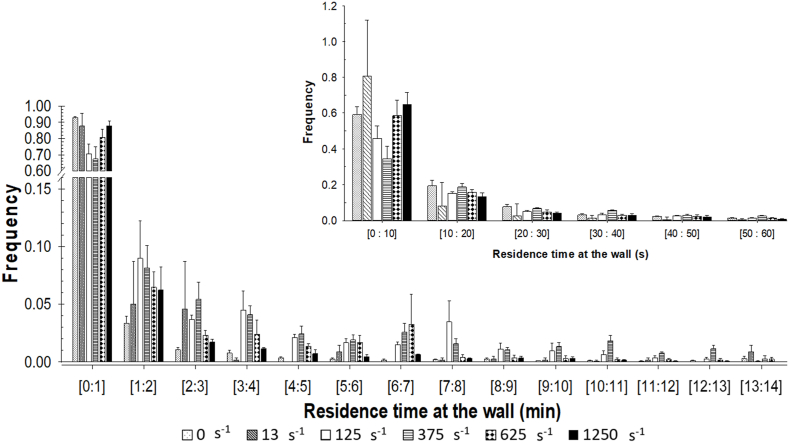
Distribution of the residence times of *S. oneidensis* at the top wall as a function of shear rate during the initial 14 min of experiment. The number of total bacteria analyzed was n_total_ = 20,344 (with n = 6053 for ${\dot{\gamma }}_{w}$ = 0 s^−1^; n = 674 for ${\dot{\gamma }}_{w}$ = 13 s^−1^; n = 3182 for ${\dot{\gamma }}_{w}$ = 125 s^−1^; n = 1801 for ${\dot{\gamma }}_{w}$ = 375 s^−1^; n = 6151 for ${\dot{\gamma }}_{w}$ = 625 s^−1^; n = 2483 for ${\dot{\gamma }}_{w}$ = 1250 s^−1^). Insert zoom on the residence time distribution of adhered bacteria remaining less than 60 s at the wall. Data were obtained from 3 independent experiments per shear rate (except for 13 s^−1^; n = 2).

Among the criteria accessible by deep learning approach, the dynamic of each adhered bacterium, particularly their residence times at the wall were determined (Fig. 6). Two distinct bacterial wall behaviors are identified, a dynamic behavior with a very short residence time and a sedentary behavior with longer adhered times. Approximately 9 % of the total adhered bacteria remained at the wall for the entirety of the 14 initial minutes. Such sedentary was predominantly observed at shear rates of 125 s^−1^ and 375 s^−1^ (Fig. 6) with over 10 % of the bacteria exhibiting a residence time on the wall exceeding 5 min. This is in line with the colonization kinetics as presented in Fig. 4. This approach represents a significant advancement in the quantification of bacteria adhesion dynamics, particularly at the early stages of biofilm formation, and highlights the pivotal role of fluid mechanics in this process. Indeed, in contrast to previous shear rates, the lowest and highest shear rates tested (i.e. 0. 13 s^−1^ and 1250 s^−1^) presented a 10-fold reduction in the concentration of sedentary bacteria (>5 min) on the wall.

In contrast, a dynamic exchange of bacteria at the wall was also observed, irrespective of the shear rate applied. Indeed, over 70 % of the pioneering bacterial population remained on the wall for less than a minute, with 72 % (±5 %) of them remaining for less than 20 s (Fig. 6 insert). However colonization ultimately occurred (Fig. 4), thereby confirming that the equilibrium between transfer-induced adhesion and detachment consistently favored successful attachment.

The probability of bacterial collision efficiency is known to be controlled by on one hand the bacteria-surface interactions, i.e. the force-sensitive adhesion/detachment mechanisms [8,76,[83], [84], [85]] and on the other hand the blocking of available adhesion sites due to the amount of previously adhered bacteria [58,78]. The physicochemical basis of adhesion mechanisms demonstrates that the strength of interactions between bacteria and a substratum was increased with bacteria wall residence time. Consequently, longer residence time of bacteria led to stronger interaction forces, making it more challenging for them to dislodge from the surface [83,85,86]. The process of “bond aging maturation” occurred rapidly, in an exponential manner over time within seconds to minutes after initial contact, as previously shown. For example, the interaction forces between bacteria and the wall were observed to increase by a factor of two on average after 200 s of contact [85]. Alternatively, the probability of bacterial detachment was found to be decreased by a factor of 100 to 1000 after 1 min of contact with the wall [83]. The hypothesis that the strengthening of bond adhesion after initial contact can be governed by the rearrangement of bacterial cell surfaces (such as the unfolding of cell surface structures) has been proposed by Olsson et al. [87] and Berne et al. [9]. Additionally, the removal of bacteria and surface interfacial water facilitating the increase in the number of contact points and enhancing hydrophobic interactions, as well as the repositioning of the bacterial cell body, and the production of adhesins have been identified as potential mechanisms for this strengthening of bond adhesion [9,58,88,89].

**Fig. 7:**
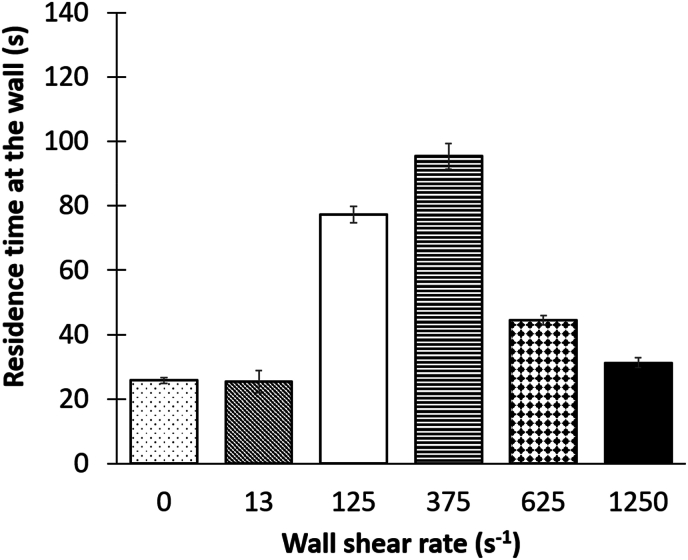
Mean residence times of adhered *S. oneidensis* at the capillary top wall during the very first 14 min of experiment as a function of shear rate applied. Histograms represent the mean of 3 independent experiments by shear rate and error bars correspond to the standard error (except for 13s^−1^; n = 2). The total bacteria number analyzed was n_total_ = 20,344 (with n = 6053 for ${\dot{\gamma }}_{w}$ = 0 s^−1^; n = 674 for ${\dot{\gamma }}_{w}$ = 13 s^−1^; n = 3182 for ${\dot{\gamma }}_{w}$ = 125 s^−1^; n = 1801 for ${\dot{\gamma }}_{w}$ = 375 s^−1^; n = 6151 for ${\dot{\gamma }}_{w}$ = 625 s^−1^; n = 2483 for ${\dot{\gamma }}_{w}$ = 1250 s^−1^).

Average residence times of pioneer bacteria on the capillary wall are comprised between a range of 25to 95 s, depending on the shear rates (with significant difference between shears: p value < 0.001) and show a bell curve profile (Fig. 7) with a wall residence time from 2 to 4 longer for 375 s^− 1^ and the 125 s^−1^ compared to the other shears. To our knowledge, only one study has investigated the influence of shear rate on the wall residence time of pioneer bacteria [22]. Although working with different experimental conditions (system, strain), their study yielded comparable insights to ours, namely a relatively low average wall residence time (between 2 and 40 s) and a bell curve profile with an intermediate shear wall existence (close to 3000 s^−1^) that maximized the bacterial residence time at the wall. Several hypotheses can be proposed to explain these observations. For shear rates between 0 and 375 s^−1^, where bacterial residence time is enhanced with increasing shear rate, it can be postulated that the interaction forces developed between bacteria and the wall were similarly improved with increasing shear rate, allowing for better anchoring to the surface, resulting in longer residence times. Such strategy suggests that bacteria are capable of sensing mechanical stress and modulating their cellular activity in response, thereby allowing them to adhere more strongly and to remain at the wall [90]). This shear adaptation mechanism has been described for several bacterial strains, including *E. coli* [91], *S. aureus* [92], *P. aeruginosa* [93] where the regulatory mechanisms involved included the production of di-GMPc [93] and adhesines [9] or even the “fro” (“flow-responsive”) operon recently discovered by Sanfilippo et al. [94].

Beyond this shear optimum, we observed that further increases in shear rates have the opposite effect on bacterial colonization (Fig. 7) revealing that the shear is too high for the bacteria to withstand and adapt to, leading to high bacterial detachment [10,11,22,46,83]. Moreover, given that wall transfer is enhanced at high shear rates (i.e. 1250 s^−1^), we can hypothesize that the number of collisions between newly transferred bacteria and those already adhered could also be greater, which could explain the stronger exchange dynamics observed at the wall, with a large number of bacteria reaching the wall, but rapidly dislodged by the following ones arriving at the wall.

### Bacterial orientation at the wall

**Fig. 8:**
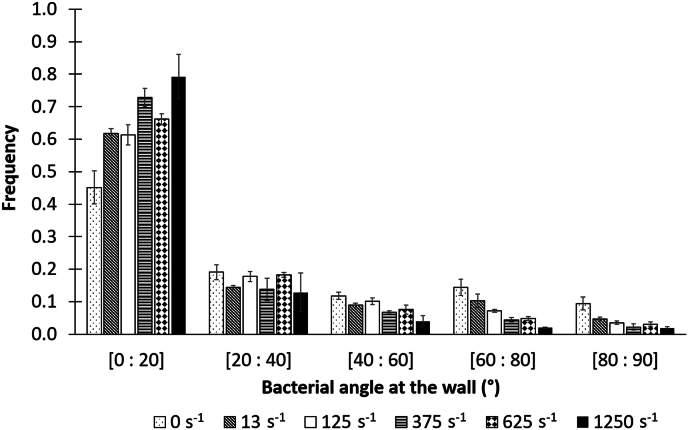
Orientation of adhered *S. oneidensis* relative to the flow direction (i.e. 0° when bacteria aligned to the flow) during the first 14 min of experiment and according to shear rate. The number of bacteria analyzed according to shear were n = 6053 for ${\dot{\gamma }}_{w}$ = 0 s^−1^; n = 674 for ${\dot{\gamma }}_{w}$ = 13 s^−1^; n = 3182 for ${\dot{\gamma }}_{w}$ = 125 s^−1^; n = 1801 for ${\dot{\gamma }}_{w}$ = 375 s^−1^; n = 6151 for ${\dot{\gamma }}_{w}$ = 625 s^−1^; n = 2483 for ${\dot{\gamma }}_{w}$ = 1250 s^−1^ (n_total_ = 20,344 bacteria). Data were obtained from 3 independent experiments per shear rate (except for 13 s^−1^; n = 2).

Given that flow is ubiquitous and that the spatial distribution of adhered bacteria is a crucial factor in the structuration of nascent biofilms [1,62], we are interested in the orientation adopted by bacteria at the wall in the presence of hydrodynamic constraints (Fig. 8). To achieve this, a mean wall angle was assigned to each bacterium by averaging all the angles taken by the bacteria at the wall during its entire residence time. Most pioneer adhered bacteria were aligned similar or very close to the direction of flow [0°; 20°]: 45 % under static condition and more than 61 % when flows were applied, with a maximum (79 %) reached at the highest shear rate. As the shear rate is increased, frequencies dropped drastically, so that the [80°; 90°] class was weakly represented (3.1 % ± 0.5 under flow flux and 9.5 % under static condition) (Fig. 8). We also noted that the fractions of bacteria with an average angle greater than 40° decreased as the shear rate increased in such a way that only 7.9 % of the bacteria oriented themselves at an angle greater than 40° to the flow at ${\dot{\gamma }}_{w}=$ 1250 s^−1^ shear rate, whereas for the other shear rates tested (excluding the static condition), this fraction represents 18.4 % (±2.3).

Already observed, but in a qualitative way [44,95,96], our study provides a robust quantification (more than 20,344 bacteria analyzed) of the orientation of adhered bacteria, revealing in particular that under flow, bacteria tend to orient themselves mainly in the same direction as the flow. An earlier study [97] on a sample of around 1000 bacteria showed the same trend. They hypothesized that the orientation of the bacteria at the wall could limit their surface area in contact with flow, thereby minimizing the mechanical stress to which the bacteria are exposed. Whether this orientation at the wall is the result of the bacteria maintaining the orientation they took in the flow (for the same reasons as above), or a reorientation of the bacteria after adhesion, remains an open question. This could be achievable by 3D imaging as for instance holographic microscopy, an emerging imaging technique [98] that allows instantaneous sampling of 3D volume and has already proven its worth for bulk bacterial tracking, motility and trajectories characterization [[99], [100]][99], [100]. To gain a more detailed understanding of what is happening at the wall, the distribution of mean angles is compared with the distribution of initial and final angles for each shear rate (Fig. S5). Results show no statistically significant differences between the distribution of initial and final angles. On the other hand, except for ${\dot{\gamma }}_{w}$ = 13 s^− 1^, the distribution of the mean angle is statistically weaker (on average 4° less) than that of the initial and final angles. This may mean a slight reorientation of the bacteria at the wall during their wall residence time, and perhaps the existence of a slight imbalance that occurs just before bacterial detachment, which reorients the bacteria at an angle (similar to that of attachment), leading to their detachment. But this is very speculative.

## Conclusion

The influence of shear rate on the dynamic of pioneer bacteria was studied through standard and deep learning approaches. A global analysis of the population based on time-lapse imaging and manual counting revealed the existence of two distinct phases in the colonization kinetics of pioneering *S. oneidensis* bacteria. Our findings suggest that the level and the rate of colonization were not inherently correlated with increases in hydrodynamic stress. Moreover, in our experimental capillary model, the mechanical mechanisms governing the bacterial wall transfer were dependent on the shear rates. Convection was identified as the primary mechanism at high shear rates. In contrast at low shear rates, sedimentation can occur in such confined system and balance convection, leading to a more heterogeneous colonization.

At the individual scale, the innovative implementation of a deep-learning method offers a significant added value, as it enables the detection and tracking at the wall of each individual pioneer bacterium (>20,300 bacteria), thus enabling the determination of each adhered bacteria's fate. This approach facilitated a comprehensive evaluation of the dynamics of just adhered bacteria, as well as the quantification of associate parameters such as the residence time of bacteria at the wall and their orientation as a function of shear rates. Our findings demonstrated that the adhered bacteria exhibited both dynamic and sedentary behaviors at the wall. Notably, over 70 % of the bacteria remained at the wall for less than a minute. The mean bacterial residence time ranged from 25 to 95 s, depending on the shears. Furthermore, the impact of shear rates on bacterial residence time yielded a bell-shaped curve, indicating the existence of a critical shear rate that enhances bacterial residence time. This finding highlights the dual impact of wall shear rates on bacterial colonization. Furthermore, our findings revealed that over 60 % of the bacteria subjected to hydrodynamic forces exhibited a parallel orientation to the flow. Such deep learning approach also provides a means of more robustly characterizing the bacterial absorption/desorption dynamics at the wall, which can be valuable for developing quantitative models of bacterial adhesion and testing transfer mechanisms.

## CRediT authorship contribution statement

**Lucie Klopffer:** Writing – review & editing, Writing – original draft, Visualization, Validation, Methodology, Investigation, Formal analysis, Conceptualization. **Nicolas Louvet:** Writing – review & editing, Validation, Supervision, Software, Project administration, Methodology, Funding acquisition, Formal analysis, Conceptualization. **Simon Becker:** Writing – review & editing, Software, Data curation. **Jérémy Fix:** Writing – review & editing, Software, Data curation. **Cédric Pradalier:** Writing – review & editing, Software, Methodology, Data curation. **Laurence Mathieu:** Writing – review & editing, Writing – original draft, Validation, Supervision, Project administration, Methodology, Funding acquisition, Formal analysis, Conceptualization.

## Declaration of competing interest

The authors declare that they have no known competing financial interests or personal relationships that could have appeared to influence the work reported in this paper.

## Data Availability

Data will be made available on request.
