# Supplementary material for: Effect of shear rate on early Shewanella oneidensis adhesion dynamics monitored by deep learning
Source: Biofilm. 2024 Nov 16;8:100240. doi: 10.1016/j.bioflm.2024.100240 (PMC11621503; doi:10.1016/j.bioflm.2024.100240)
Supplement: Multimedia component 1 [file mmc1.docx]

**Effect of shear rate on early *Shewanella oneidensis* adhesion dynamics monitored by Deep Learning**

**Appendix A. Supplementary data**

Lucie Klopffer^a,b^, Nicolas Louvet ^b*^, Simon Becker ^b^, Jérémy Fix ^c^, Cédric Pradalier ^d^, Laurence Mathieu^e*^

^a^ Université de Lorraine, CNRS, LCPME, F-54000 Nancy, France

^b^ Université de Lorraine, CNRS, LEMTA, F-54000 Nancy, France

^c^ Unviversité de Lorraine, CNRS, Centrale Supélec, F-57070 Metz, France

^d^ GeorgiaTech Europe, IRL 2958, F-57070 Metz, France

^e^ EPHE, PSL, UMR CNRS 7564, LCPME, F-54000 Nancy, France

*Corresponding authors:

- **Laurence Mathieu**

LCPME, Campus Brabois Santé, Bâtiment AB, 9 Avenue de la Forêt de Haye, BP 20199, F-54505 Vandoeuvre-lès-Nancy, France

[laurence.mathieu@univ-lorraine.fr](mailto:laurence.mathieu@univ-lorraine.fr)

- **Nicolas Louvet**

LEMTA, 2 Avenue de la Forêt de Haye, BP 90161, F-54505 Vandoeuvre-lès-Nancy, France

nicolas.louvet@univ-lorraine.fr


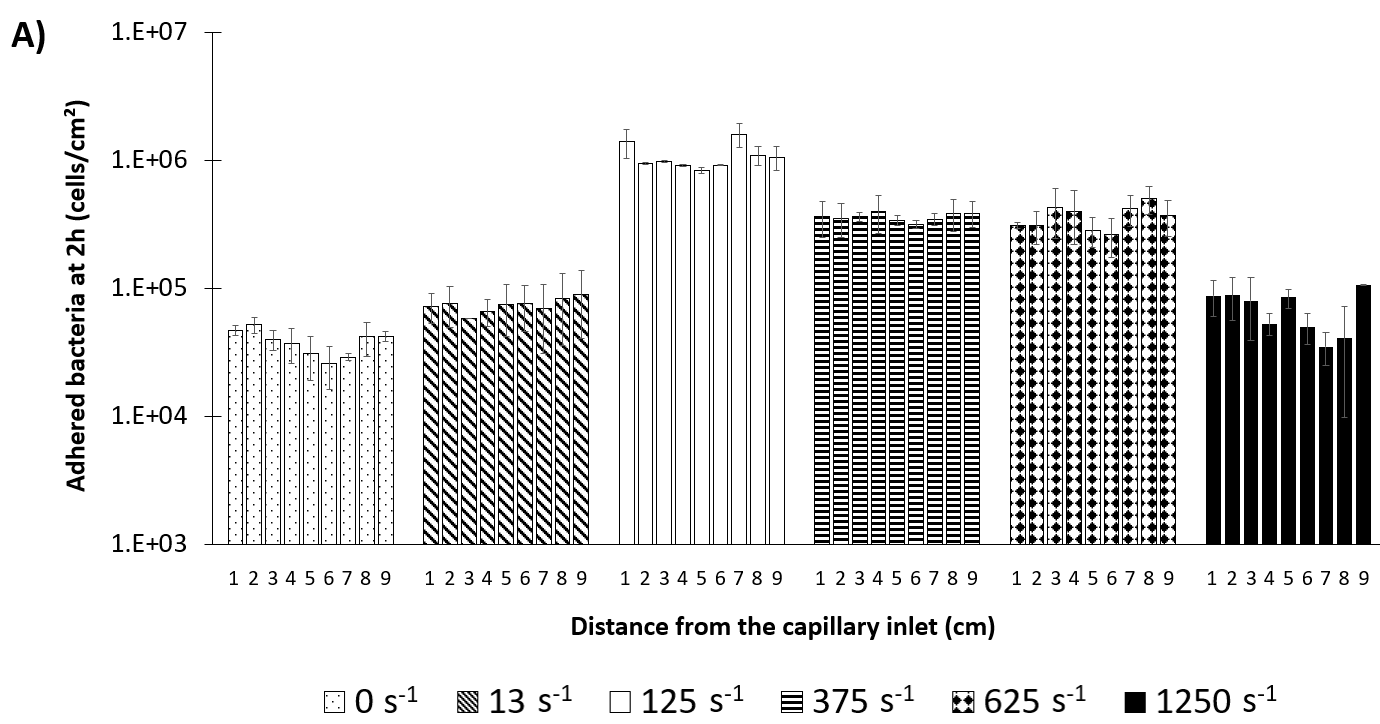

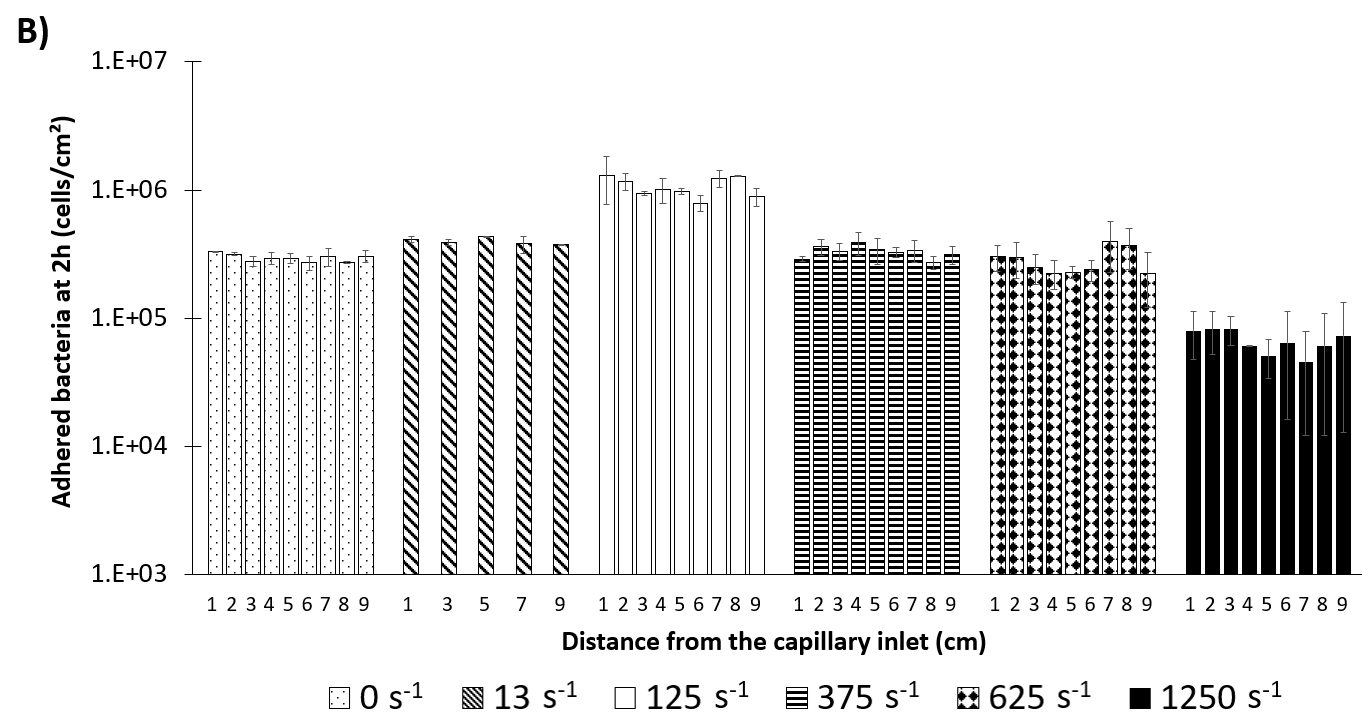


**Figure S1:** Adhered bacterial cells at the upper (A) and lower (B) capillary wall at different distances (9 distances 1 cm apart) from the capillary inlet after 2 h of colonization and for different shear rates. For each shear rate, histograms represent mean and standard error for 3 independent experiments.

| **Time(h)** | **Total biomass**  **(cells/mL)** | **Membrane damaged bacteria (PI^+^) (cells/mL)** | **Percentage of bacteria PI^+^ (%)** | **Culturable bacterial cell**  **(CFU/mL)** | **Percentage of culturable bacteria (%)** |
| --- | --- | --- | --- | --- | --- |
| **0** | 4.8 × 10^7^ ± 4.5 × 10^6^ | 9.7 × 10^5^ ± 3.8 × 10^5^ | 2.0 ± 0.6 | 3.3 × 10^6^ ± 7.3 × 10^5^ | 9.0 ± 0.7 |
| **2** | 3.4 × 10^7^ ± 5.2 × 10^6^ | 2.8 × 10^5^ ± 8.2 × 10^4^ | 1.0 ± 0.3 | 3.4 × 10^6^ ± 9.5 × 10^5^ | 7.1 ± 1.8 |

**Table S1:** Bacterial inoculum characteristics: number of total, damaged membrane and culturable bacterial cells in the feeding medium (planktonic form) at the beginning (t = 0h) and at the end of the bacterial adhesion’s phase (t = 2h). All the data represent mean and standard error from 5 independent experiments.

For culturability, 10 µL from multiple 10-fold dilutions of the bacterial suspension were plated on LB_1/10_ agar media and after incubation of 2 days at 30°C, the number of colonies were counted and results expressed as CFU per milliliter.

For total bacteria and damaged membrane bacteria counting, the GFP-Shewanella strain was stained with a propidium iodide solution (PI, B34954, Invitrogen, 20 µM final concentration) for 15 minutes in the dark. The quantification of both total and damaged biomass was conducted using the BD Accuri C6 Plus flow cytometer (BD Biosciences, USA), which was equipped with a blue laser at 488 nm. Ultrapure water (OTEC Aguettant, France) was utilized as the sheath fluid, and all data were acquired over a two-minute period at a flow rate of 35 µL/min. The total biomass, which corresponded to the GFP-tagged bacteria, was detected using the FL1 detector, with a wavelength of 533 nm ± 30. The damaged-membrane bacteria were determined using the FL2 detector (585 nm ± 40). The events were triggered on the forward scatter (FSC) parameter with a threshold of 2500 and on the FL1 detector with a threshold of 700 for total biomass. The data were analyzed using the BD Accuri™ C6 software (BD Biosciences).

In average, the cultivable fraction represented approximately 10% of the total biomass, while the proportion of damaged membranes fell below 2%.  **Supplementary data S1**

In order to differentiate motility effect from the fluid convection as a mechanism to reach the wall, we compare the characteristic time-scale for a bacterium to be align by the shear (‘*t_shear_*’) with the characteristic time for a bacterium to swim across the channel height (‘*t_swim_*’).

The time-scale *t_shear_* is evaluated as the inverse of the wall shear rate : t_shear_ ≈ $1/{\dot{\gamma}_{w}}$. Assuming that a bacterium swims at a constant velocity *V_0_*, the time *t_swim_* is evaluated as *t_swim_* $\approx h/{(2*V0)}$ with h the channel height.

The ratio between these two time-scales leads to $\frac{tswim}{tshear}=\frac{\dot{\gamma}_{w}*h}{2*V0}\approx30$with $\dot{\gamma}_{w}$ = 13s^-1^ for the lower flow rate, and assuming an average bacterium velocity *V_0_* $\approx$ 40 µm/s and *h* = 200 µm. It means that a bacterium aligns with the flow much faster than it can cross the streamlines.

In this analysis we have neglected the rotational diffusion coefficient of the bacterium that models the tumble dynamic. From visual inspection of our *Shewanella oneidensis* strain we assume that it behaves like a smooth swimmer and is able to swim with a persistent length much larger than ten times its body size. This is coherent with a value of the rotational diffusion coefficient *Dr* ≈ 0.5 - 1 rad²/s. We can also compare the rotational diffusion time with *t_shear_* which is nothing less than the Peclet number *Pe* = ${\dot{\gamma}_{w}}/{Dr.}$Again for the lower shear rate we evaluate the Peclet number around 10 meaning that rotational diffusion can be neglected.

In fact, for low ratio *t_swim_*/*t_shear_*, bacterial motility does indeed improve the transfer of bacteria to the wall and consequently the efficiency of bacterial adhesion, as shown in several studies that investigated bacterial motility as a means of accessing the wall [1–6]. However, with increasing flow and shear rate, these studies also showed that the resulting colonization becomes similar in the presence of motile and non-motile populations, suggesting that transfer to the wall by motility becomes insignificant. Some studies go even further [7,8] and hypothesize that bacterial motility is inhibited at high shear rates due to the shear forces that would cause deterioration, blockage or even breakage of the bacterial flagellum.

[1] D.R. Korber, J.R. Lawrence, B. Sutton, D.E. Caldwell, Effect of laminar flow velocity on the kinetics of surface recolonization by Mot+ and Mot− Pseudomonas fluorescens, Microb. Ecol. 18 (1989) 1–19. https://doi.org/10.1007/BF02011692.

[2] T. Kaya, H. Koser, Direct Upstream Motility in Escherichia coli, Biophys. J. 102 (2012) 1514–1523. https://doi.org/10.1016/j.bpj.2012.03.001.

[3] J.W. McClaine, R.M. Ford, Characterizing the adhesion of motile and non motile Escherichia coli to a glass surface using a parallel-plate flow chamber, Biotechnol. Bioeng. 78 (2002) 179–189. https://doi.org/10.1002/bit.10192.

[4] E. Secchi, G. Savorana, A. Vitale, L. Eberl, R. Stocker, R. Rusconi, The structural role of bacterial eDNA in the formation of biofilm streamers, Proc. Natl. Acad. Sci. 119 (2022) e2113723119. https://doi.org/10.1073/pnas.2113723119.

[5] S. Zheng, M. Bawazir, A. Dhall, H.-E. Kim, L. He, J. Heo, G. Hwang, Implication of Surface Properties, Bacterial Motility, and Hydrodynamic Conditions on Bacterial Surface Sensing and Their Initial Adhesion, Front. Bioeng. Biotechnol. 9 (2021) 643722. https://doi.org/10.3389/fbioe.2021.643722.

[6] B. Ezhilan, D. Saintillan, Transport of a dilute active suspension in pressure-driven channel flow, J. Fluid Mech. 777 (2015) 482–522. https://doi.org/10.1017/jfm.2015.372.

[7] M. Molaei, J. Sheng, Succeed escape: Flow shear promotes tumbling of Escherichia colinear a solid surface, Sci. Rep. 6 (2016) 35290. https://doi.org/10.1038/srep35290.

[8] J. Yang, K. Kikuchi, T. Ishikawa, High shear flow prevents bundling of bacterial flagella and induces lateral migration away from a wall, Commun. Phys. 6 (2023) 354. https://doi.org/10.1038/s42005-023-01471-y.


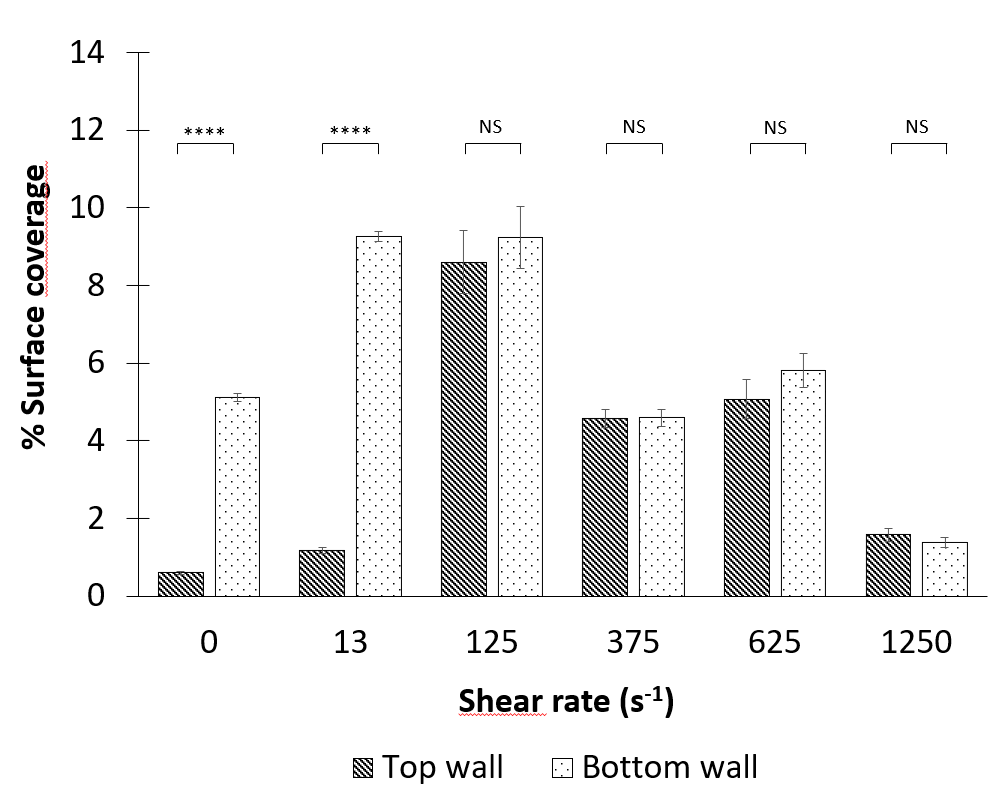


**% Surface coverage**

**Shear rate (s^-1^)**

**Figure S2:** Average surface coverage (%) of adhered *S. oneidensis* at the top and bottom wall of the capillary according to shear rates after 2h colonization. For each shear rate, histograms represent mean and standard error for 30 independent values per shear rate and per wall condition. (NS = not statistically significant, **** = p value < 0.0001).

**Figure S3**: Representative epifluorescence microscopic observations of adhered *S. oneidensis* gfp cells at the top wall capillary for 2 shear rates: 125 s^-1^ (a,b,c,d) and 1250 s^-1^ (e,f,g,h).and at 4 colonization times : 0.08h (a,e) ; 0.5h (b,f) ; 1h (c,g) ; 2h (d,h). Flow is right to left. Scale bar represents 100 µm.


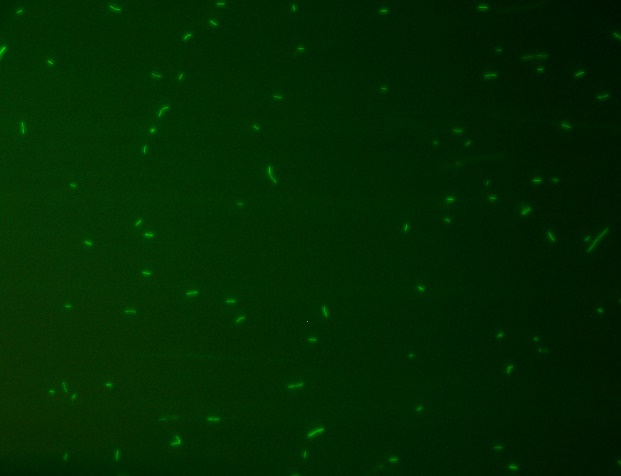

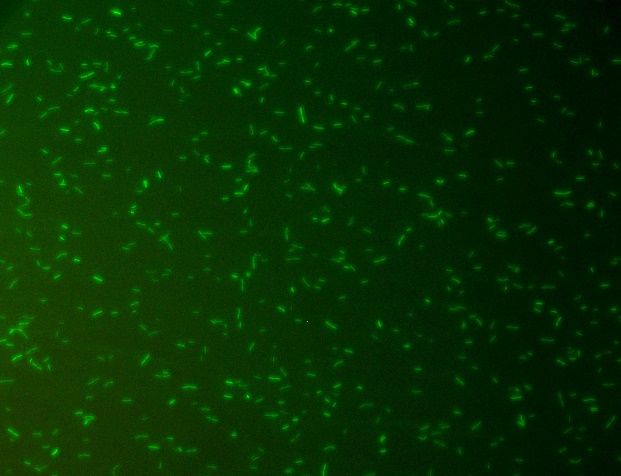

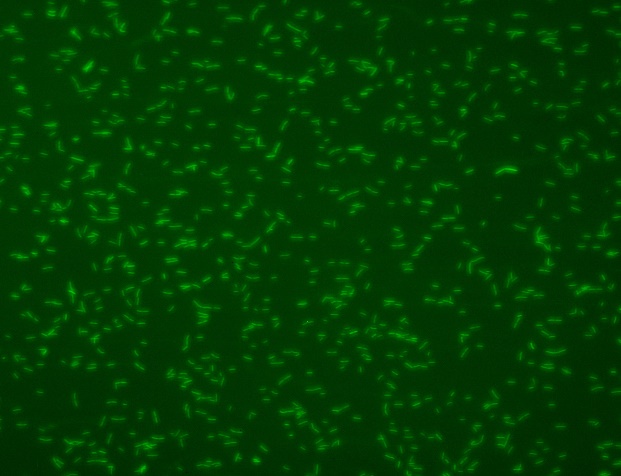

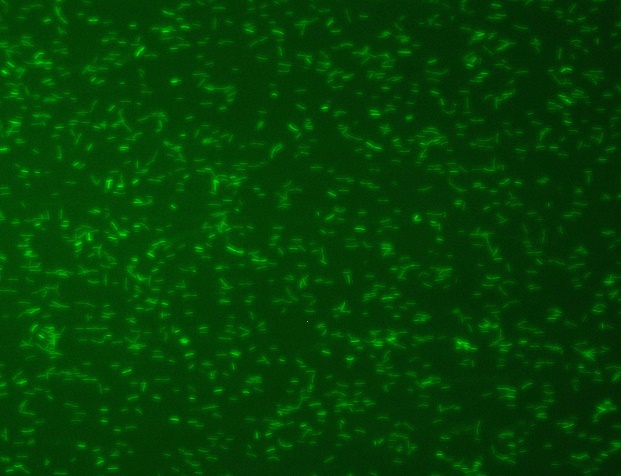

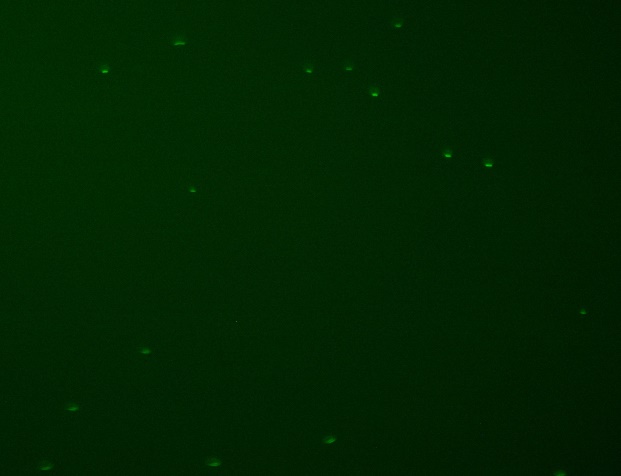

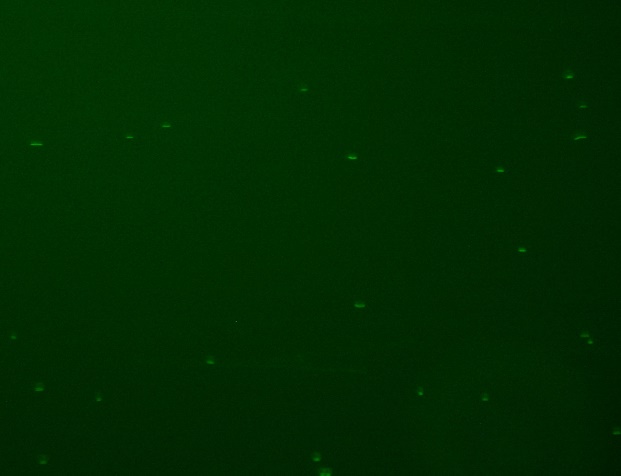

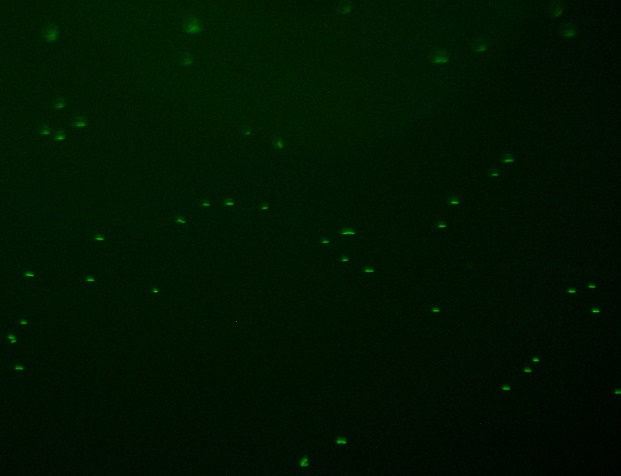

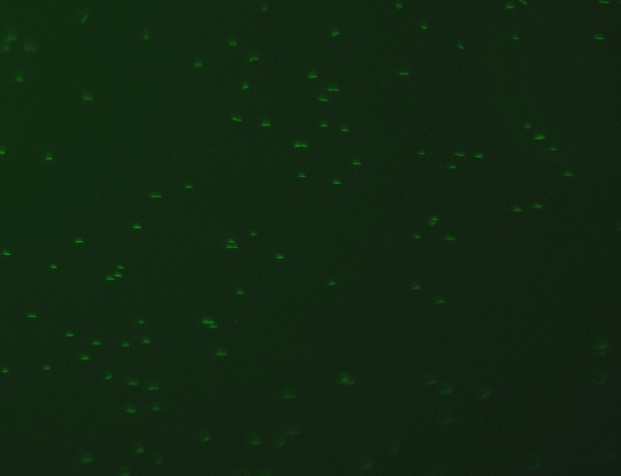


a

b

c

d

h

g

f

e

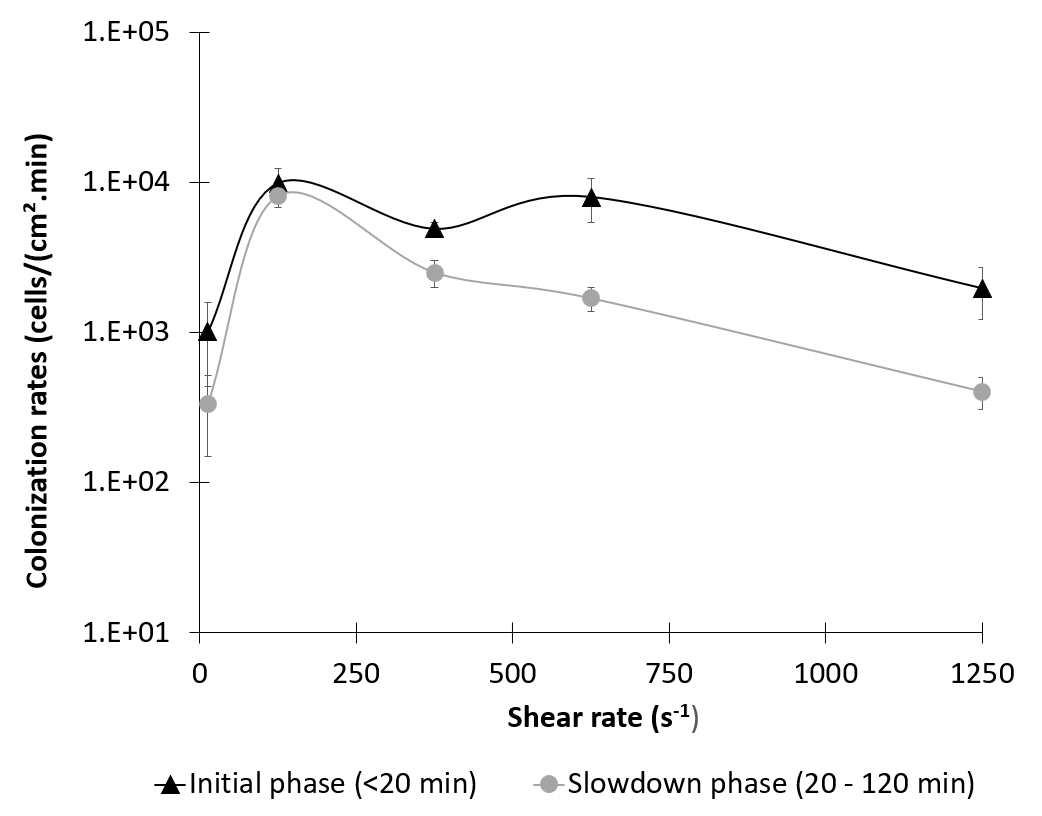


**Figure S4:** Colonization rates of *S. oneidensis* at the upper capillary wall during the first two hours of colonization as a function of shear rates. Two distinct phases were considered: the initial phase (<20 min of colonization; see Fig. 4) and the slowdown phase (20 to 120 min of colonization; see Fig. 4). Each value is the mean and standard error of three independent experiments. The curves are for display purposes only.


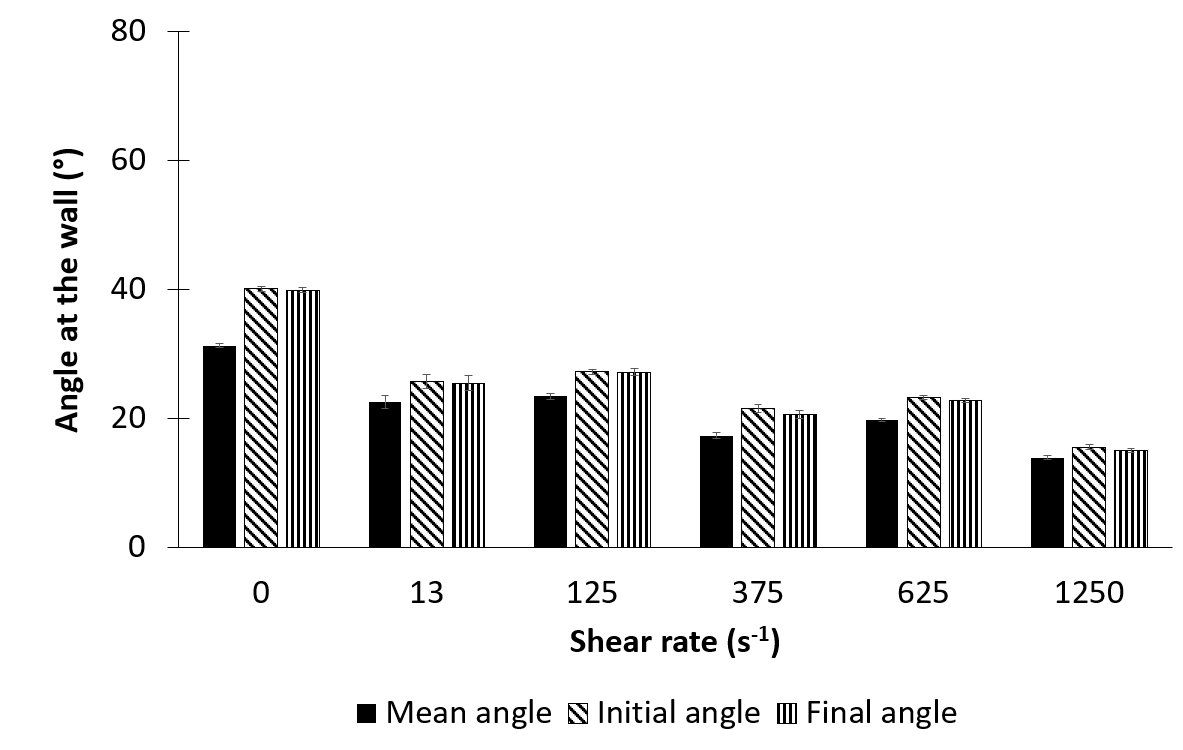


**Figure S5:** Mean, initial and final wall orientation, relative to the flow direction of *S. oneidensis* adhered bacteria during the first 14 min according to shear rate. The total number of bacteria analysis was n_total_ = 20,344 bacteria (with n = 6,053 for $\dot{\gamma}_{w}=$0 s^-1^; n = 674 for $\dot{\gamma}_{w}=$13 s^-1^; n = 3,182 for $\dot{\gamma}_{w}=$125 s^-1^; n = 1,801 for $\dot{\gamma}_{w}=$375 s^-1^; n = 6,151 for $\dot{\gamma}_{w}=$625 s^-1^; n = 2,483 for $\dot{\gamma}_{w}=$1,250 s^-1^). Data obtained came from 3 independent experiments by shear rate (except for 13 s^-1^; n = 2).
